# Immersive Experience Model of the Elderly Welfare Centers Supporting Successful Aging

**DOI:** 10.3389/fpsyg.2020.00008

**Published:** 2020-02-11

**Authors:** Eun J. Lee, Sung J. Park

**Affiliations:** ^1^Department of Architecture, Keimyung University, Daegu, South Korea; ^2^Department of Architectural Engineering, Keimyung University, Daegu, South Korea

**Keywords:** successful aging, elderly welfare center, the elderly, immersive experience, immersive experience service, immersive technology

## Abstract

This study investigates the application of immersive experience services to leisure facilities for the successful aging of the elderly. In the past, the social image of the elderly was that of passive beings who needed care due to physical and cognitive decline associated with biological aging. However, the concept of “successful aging” actively highlighting the positive aspects of aging and trying to promote longer and healthier life has started to acquire importance in recent years. In this context, elderly welfare centers can be described as facilities that encourage learning, leisure, and social activities of the elderly with impaired physical and cognitive functions. The use of recent immersive experience technologies such as virtual reality and mixed reality (MR), in order to mitigate physical and spatial constraints and provide an immersion into the desired environment and situation, could contribute substantially to the health of the elderly. However, the application of immersive technologies is concentrated on the provision of entertainment, education, and medical facilities. The number of previous studies on the immersion experiences of the elderly is limited, and the connection between immersion experiences and various services and programs that promote successful aging at elderly welfare centers requires further research. This study analyzes the function and space of the elderly welfare centers for successful aging through the review of previous studies and classifies immersion technology categories based on the review of the relevant literature. The study analyzes the health benefits of immersive experience technologies and related products and services and proposes an immersive experience service model for the elderly welfare center. The results of the study could provide a valuable input for the spatial application of immersive experience technologies for successful aging in the future.

## Introduction

The rapid growth of the elderly population is a global trend. Korea has become an aged society in 2017, after only 17 years as an aging society. The proportion of people aged 65 or older currently exceeds 15 percent, with the elderly population expected to reach 10 million people by 2026, making Korea a post-aged society (Statistics Korea, 2018). Korea’s entry rate to aging society is considerably faster than in other developed countries such as the United States (73 years), Germany (40 years), and Japan (24 years) (OECD, 2005). Population aging is generally perceived as an increase in older (over 65) dependent populations that need to be cared for financially and medically in terms of national and social aspects. However, with the continued improvement of the educational and economic levels of the elderly, there is a growing interest in “successful aging,” a term which highlights the positive aspects of aging and involves a longer and more active life. Successful aging is the process of proactively coping with the fear of inevitable declines associated with an old age; this is done by maintaining high levels of physical, mental, and social activity and acting as an active rather than passive member of society. This new perception of aging requires a new paradigm different from the existing focus on the medical and welfare systems.

Recent studies suggest that “immersion experience” technology using information and communication technology (ICT) and virtual reality (VR) can promote the health and well-being of the elderly (Yamagami et al., 2007; Angelini et al., 2015). Immersive experience can contribute significantly to successful aging by inducing positive experience and facilitating communication of the user through a new or familiar virtual content. However, the existing immersion technology applications are largely provided for the purpose of entertainment, education, or therapy. They have limited application measures to the housing and hospital used to house and care of the elderly. The elderly welfare centers that provide leisure activities, disease prevention, and social interaction need to find ways to also provide these and other cost-effective services from the perspective of successful aging.

The purpose of this study is to develop the immersive experience service model that can be applied at the elderly welfare centers via linking the main functions of the elderly welfare center for successful aging with the immersive experience technology that can support it. This study analyzes the possibility of the positive immersive experience for the elderly and suggests an interaction and application method based on the programs supported by the elderly welfare centers as well as spatial needs. The immersive experience service model developed here suggests new developments in the field of immersive experience content and healthcare services.

## Research Scope and Methodology

**FIGURE 1:**
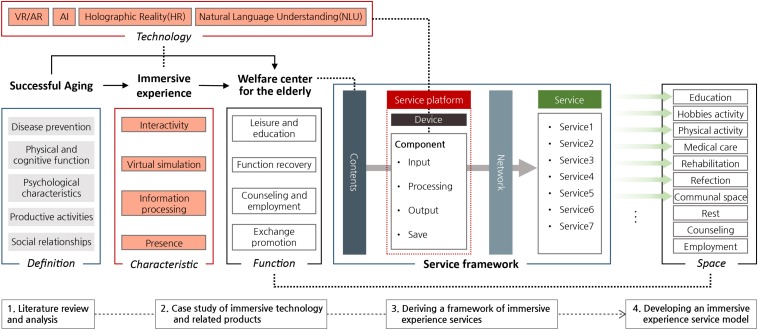
Research schematics.

Several research steps have been taken in this study (Figure 1). Firstly, the concept of successful aging and the characteristics of immersive experience were analyzed through literature review. Secondly, the study analyzed the function and program space at the elderly welfare centers from the perspective of successful aging and reviewed the literature focused on identifying the positive effects of immersive technology for the elderly. Here, we analyzed the immersive experience technology and related products and services for the elderly. The literature reviewed included journal articles, extended abstracts, and conference proceedings, using databases such as ScienceDirect, ERIC, SpringerLink, and EMBASE. Three search keywords were used: “elderly,” “immersive technology,” and “immersive experience.”

Thirdly, we derived service contents and related immersion skills based on the characteristics of the immersion experience and the function of the elderly welfare center. Fourthly, the immersive experience service composition system and a service platform were established, and the contents of the immersive experience service-based service platform were derived based on the functions of the elderly welfare centers. And finally, an immersive experience service model is proposed, taking into account the spatial function of the elderly welfare centers.

## Successful Aging and Immersive Experience

### Successful Aging

“Aging” is a progressive degeneration process that is accompanied by a decline in biological function and an increase in mortality with age (Hayflick, 2000). With the development of medical technology and educational level, elderly health care has begun to focus on understanding not only biological aging but also social and psychological aging and on seeking ways to support the multidimensional needs and requirements of the elderly. In particular, as social myths about aging gradually shift from negative to positive, “successful aging” has emerged as the subject of neo-aging and has been studied in various definitions and measures over the past decades (Rowe and Kahn, 1998; Nusselder and Peeters, 2006; Ouwehand et al., 2007; Chodzko-Zajko et al., 2009). A theory of successful aging has been established to describe conditions meeting social and biomedical needs of the elderly. The former includes satisfaction with life, psychological well-being, social functioning, and so forth, whereas the latter emphasizes physical and cognitive functionality. Havighurst (1963) defined successful aging as social adaptation from a social psychology perspective, stating its importance for social stability and the flexibility of personality and relationships in this process. On a social side, successful aging implies that emotional attachment and self-esteem stem from personal experiences and memories, controllable circumstances, or circumstances not necessarily influenced by environmental characteristics (Baltes and Baltes, 1990) and that the relaxation of mind through involvement with nature is one of the main factors of being happy in old age (Bhatt, 2015). Successful aging on the biomedical side, on the other hand, implies minimizing the physiological degradation and increasing active life expectations through the prevention of chronic diseases (Chodzko-Zajko et al., 2009). Other successful aging factors include longevity, active (healthy) life, disease-free life, absence of chronic disease, reduced depression and loneliness, and high physical and mental functionality (Nusselder and Peeters, 2006; Ouwehand et al., 2007).

In contrast, Rowe and Kahn (1998) noted that aging should be considered in a variety of ways, rather than in one aspect. This can be explained by distinguishing between the usual aging and successful aging. Usual aging refers to helpless old age with no specific disease or dysfunction, but with risk factors for disease, while successful aging can overcome or delay the universal aging with appropriate control of the environment and individual efforts. Thus, aging may be different depending on the environment or individual ability; therefore, supportability should be considered in individual and diversified aspects.

Recent studies have categorized the concept of successful aging into five different areas, namely, disease prevention and health, physical and cognitive functioning, psychological characteristics, social relations, and productive activities (Rowe and Kahn, 2015). Learning is often emphasized as a successful aging factor (Duay and Bryan, 2006), and research shows that participation in educational and creative activities in old age can have a positive impact on aging (Gallistl, 2018). The purpose of this study is to support successful aging in the five categories listed above. These are described below in more detail (Table 1).

**TABLE 1:** Definition of successful aging.

Successful aging	Definition
Disease prevention	Prevention of disease-related hazards, knowledge of health, and awareness of one’s health condition
Physical and cognitive function	High physical and mental functions, high learning ability and short-term memory, proper exercise, and physical fitness training
Psychological characteristics	Emotional stability, attachment to experiences and memories, absence of depression and loneliness, high degree of self-respect and satisfaction with life
Productive activities	Continuing educational and self-development, motivation for life, social activities, and an active attitude to learning
Social interaction	Close relationships with others, forming social bonds, adaptability to the environment, and social support

Disease prevention is the prevention of risk factors for diseases and disorders. Preventive support is particularly important for older people because they may experience chronic or non-specific illnesses including multiple medically unexplained symptoms (MMUS). Physical and cognitive functioning describes the potential for the elderly people to live independently and is also a prerequisite for successful aging. The psychological characteristics involve acknowledging aging and embracing various aspects of one’s life, looking forward to life, and finding a meaning. Productive activities include a positive view of knowledge and skills and, in particular, educational activities that, along with self-development, can be a motivation for a new life goal (Vaillant, 2008). Finally, maintaining social relationships with others is related to the adaptability to the environment and provides an opportunity for an improved well-being. In other words, successful aging should strive to expand the individual’s capacity and help maintain better functions, as well as to support active relationships and participation in productive roles rather than adapting to the existing society.

### Characteristics of Immersive Experience

Recent advances in media technology have resulted in the development of the new display and communication devices that can simulate environments which are very similar to reality or simulate a complete virtual reality (Jeong, 2019). The user can then maximize their presence and immersion in the displayed environment and experience a natural and intuitive interaction with the 3D structure. The result is an immersive user experience and improved interaction technologies that provide an opportunity to better serve the elderly who are vulnerable to the information society and digital devices. The ultracompact devices or smart devices that are required in modern society cannot meet the physical, cognitive, and emotional needs of the elderly due to the small displays and the need for continuous operation. Immersive technology supports 3D virtual content and natural communication and helps to improve social interactions and physical functions by allowing the user to be immersed in the desired environment (Whitlock et al., 2012; Wiemeyer and Kliem, 2012; Wu et al., 2013).

Immersion means lifelike vividness of the user surroundings and provides various immersion experiences by maximizing reality and minimizing physical constraints. The immersive experience can display characteristics such as interactivity, virtual simulation, information processing, and virtual presence. The details of the immersion experience characteristics are shown in Table 2 (Jeong, 2019).

**TABLE 2:** Characteristics of immersive experience and technology (based on study by Jeong, 2019).

Characteristics of immersive experience	Contents
Interactivity	• Users can quickly and easily select the information they want and can control or adjust the situation based on the information they choose
Virtual simulation	• Users can reproduce or manipulate things that are not directly experienced within a complete virtual space • Users can communicate through an “avatar” with other participants in the virtual space
Information processing	• Various sensory sensors can acquire and transmit information about the user • A kind of sensory immersion, awareness of the user’s biological signals and behaviors, and automated service delivery
Presence	• Users can experience mixed reality (MR) and realistic media in realistic three-dimensional and vivid expression • Includes super-high-definition display and holograms, audio system, etc.

Immersive experience promotes user-centered information transfer and exchange while leading to satisfactory results by allowing interaction with the environment as an active agent (Sauter, 2003). In other words, it satisfies the desire for empathy going beyond the sensory stimulation of the individual and has a positive effect on cooperativeness and social exchange. As a result, immersive experience has the potential to become a tool for achieving a physical, emotional, and social satisfaction. Based on all of this, it is essential to discuss the use of immersive experience in the context of health and welfare services provision.

## Elderly Welfare Centers and Immersive Technology

### Elderly Welfare Center Supporting Successful Aging

The elderly welfare centers in Korea are leisure facilities for the elderly, providing leisure activities, health care, and social activities for the elderly. They are similar to the elderly welfare centers in Japan and the multipurpose senior centers in the United States, as well as Altentagesstätta in Germany (Lim et al., 2014). The goal of the elderly welfare centers is to provide a comprehensive service for the elderly in different communities, for them to be able to live comfortable and independent lives (Park et al., 2013). To this end, the Elderly Welfare Act proposes operating and facility standards for these centers through the Elderly Welfare Center Enforcement Rules. The operation of the elderly welfare centers is defined as a provision of life and health counseling for the elderly, provision of employment information, rehabilitation or functional reinforcement training, and provision of programs to improve education including liberal arts. Additional detailed programs and services should be planned, taking into account the characteristics of each community and audience.

The elderly welfare centers are a community-oriented elderly welfare service network supporting the creation of local communities and the multidimensional welfare of the elderly following the current direction of elderly welfare practice which agrees with the “aging in place” (AIP) concept. AIP is a strategy for successful and active aging, meaning aging with people who are familiar in familiar areas. However, this strategy is also used in the expectation that community care is cost-effective in mitigating financial burdens (Vasunilashorn et al., 2012). Accordingly, this study found similarities between the direction of successful aging and the ultimate goal of the elderly welfare centers. The functions of the elderly welfare centers were categorized into leisure and education, function recovery, counseling and employment, and social interaction promotion functions.

An elderly welfare center usually has a floor area of 500 m^2^ or more and at least one essential facility such as an auditorium, an office, a counselor office, a restaurant, or a program room. As of 2018, there were 385 elderly welfare centers in Korea, with an average total floor area of 2,500 m^2^, which is about three times larger than that in Japan (Ministry of Health and Welfare, 2019). Compared to other elderly leisure welfare facilities, the ratio is high in scope, number, and number of users. However, since the 2011 amendment to the Elderly Welfare Act, there have been no significant changes in the provision of the programs in these centers (Hong and Yoo, 2017).

This study derives spatial composition based on the function of elderly welfare centers for successful aging (Park et al., 2013; Shin, 2016; Hong and Yoo, 2017; Kim et al., 2017). Table 3 summarizes the association between the function of an elderly welfare center and successful aging. Depending on the function, an elderly welfare center can consist of up to 10 spaces for hobbies, physical activities, education, medical care, rehabilitation, counseling and employment, refection, and rest. Each space is classified into a detailed set of functions, based on the intended program of use. Based on the analysis of the spaces related to successful aging, we found an association between disease prevention (5), physical and cognitive functions (7), psychological characteristics (4), and productive activities (3) and social relationships (5).

**TABLE 3:** Functions and space composition of the elderly welfare center for successful aging.

Welfare facility for the elderly			Successful aging				
				Physical and			
			Disease	cognitive	Psychological	Productive	Social
Function	Space		prevention	function	characteristics	activities	relationships
Leisure and education	Hobbies activity	Multipurpose room, auditorium, hobby classroom, arcade/game room, art gallery	•	•		•	•
	Physical activity	Weight room, yoga/aerobics room, sports room	•	•			
	Education	Classroom, computer room, library, assembly hall		•		•	
Function recovery	Medical care	Examining room, physiotherapy room, Chinese medical treatment room	•	•	•		
	Rehabilitation	Movement training room, rehabilitation room, meditation room, recovery room	•	•	•		
Counseling and employment	Counseling	Health counseling and guidance room, psychology consultation room	•	•	•		•
	Employment	Employment consultation room, employment information room				•	
Exchange promotion	Refection	Restaurant, kitchen, cafeteria					•
	Rest	Indoor garden, lounge			•		•
	Communal	Entrance, stair hall, corridor, lobby		•			•
	space						

*“∙” means there is a connection between rows and columns.*

### Immersive Experience Technology for the Elderly

A recent innovative immersion technology implementation included sophisticated computer graphic technology, network technology for massive data transmission, and a human-centered interaction solution that delivers five senses (Cummings and Bailenson, 2016). Immersion technology is a computer-based device to improve user attention and concentration, in both physical and cognitive respects. It is made by combining technologies from various fields (Keshner and Kenyon, 2004). More specifically, immersive experience technology includes technologies supporting multimodal interaction to immerse the user in a virtual platform which is the result of a computer combined with the real physical environment. This study analyzed the characteristics of the immersive experiment and contents related to immersion technology through advanced research on technology for the elderly (Table 4). Previous studies focused on analyzing the elderly’s interaction with virtual information and objects or techniques for immersing in information and content provided. Experimental studies related to the immersion experience of the elderly mainly focus on the contents of the Internet and smartphone use. There is insufficient empirical research on the immersion technology utilization and immersion experience. Immersion technology and immersion experience for the elderly can be classified into interactive video game-based (IVGB) games, robots based on artificial intelligence (AI) or natural language understanding (NLU), and immersive content using augmented and mixed reality. The results of previous studies show that immersion experiences are related to physical, mental, and social functions of the elderly, especially the IVGB training games that improve sensory and motor skills, perception, and cognition (Whitlock et al., 2012; Lai et al., 2013) and form relationships with the grandchildren (Wollersheim et al., 2010). The AI robots used for social care promote social exchange and emotional relaxation (Libin and Cohen-Mansfield, 2004; Wada and Shibata, 2006), and the use of immersive content can enhance life satisfaction and family bonds (Cornejo et al., 2013).

**TABLE 4:** Summary of previous studies on immersive experience for the elderly.

Author (year)	Participants/users	Immersive technology	Methods	Outcomes	Interrelationship		
					Phys.	Psych.	Soc.
Lai et al., 2013	30 elderly people over 65 years of age	Interactive video game-based (IVGB) physical training using the Xavix measured step system (XMSS)	Berg balance scale (BBS), timed up and go (TUG), modified falls efficacy scale (MFES) were measured before and after IVGB training	Improvement in all results based on the measurements of BBS, TUG and MFES, compared to the results before the IVGB training	•		
Whitlock et al., 2012	39 elderly aged 66–77	Conduct performance assessments of the multiplayer online WoW (War of Warcraft) game for cognitive training. In WoW, players complete quests in a persistent virtual world to receive rewards and gain levels, many of which require collaboration and social interaction with other players	Used the SPPB (Shipley institute of living scale) to perform spatial visualization measurements, perceptual rate measurements, inference ability and memory tests	Following the WoW game, participants’ cognitive abilities appeared to improve. No relationship was found between the age and cognitive change. Lower-ability elderly stood to benefit more from cognitive training		•	
Wollersheim et al., 2010	Older adult women (*n* = 11)	Wii games played included table tennis, golf, bowling, sword fighting, archery, boxing, frisbee, tennis and cycling. Standard devices were used such as Wii software, television screen, etc.	Users played Nintendo Wii Sport game twice a week for a 6-week period. Full body movements were measured using accelerometers, and psychosocial effects were assessed through end-of-study focus group meetings	Participants’ athletic level has been improved, through easy to learn Wii games. Many of the women noted that playing the games allowed them to feel more connected to their grandchildren. Overall, the results revealed that the participants perceived an improved sense of physical, social and psychological well-being	•	•	•
Anguera et al., 2013	174 participants aged 20–79	NeuroRacer is a game developed to train multi-tasking skills during driving. The game is driving in virtual reality and includes step-by-step training content that must perform various quests	Using a stepwise algorithm, the participants individually set the level of difficulty in the game. The criterion for improving multitasking ability is limited to the settlement being improved by more than 80% in the same quest	After a month of training, the results showed significant improvements in the multitasking abilities of the elderly and, in the best case, the participants reached the same level as the one they had in their 20s	•		
Banks et al., 2008	38 elderly people living in long-term facilities	Sony’s Aibo is a dog-shaped robot that includes actuators, camera and touch sensors, and stereo sound. Aibo is mobile and autonomous. It can find its power supply by itself and it is programmed to play and interact with humans	Experiments were divided into three groups: uncontrolled group A, group B that lived with Aibo, group C that lived with live pet dog. Groups B and C lived for 8 weeks, 30 min a week with Aibo or a pet dog, and during the experiments, all groups received the UCLA depression scale	Groups B and C were much less lonely than group A, and overall social satisfaction improved. No difference in results was found between having a real pet dog and Aibo		•	•
Lera et al., 2014	Five elderly people aged 59, 63, 85, 86, and 90.	Developed a prototype of an AR-based interactive robot (MYRAbot) to make it easy to use medical drug dispensers. MYRAbot communicates with the elderly in real time through cameras, speakers, communications systems, etc. The main purpose is to provide information about the time and dosage medicine intake	A small number of the elderly were tested and then questioned on their acceptability and subjective feelings for MYRAbots (Likert 5-point scale)	Overall satisfaction with social intimacy and enjoyment averaged 4.73, a very high average. In addition, the lower the age, the higher was the satisfaction score for usefulness and functionality	•		•
Wada and Shibata, 2006	12 elderly aged 67–89 in care house	Paro is a seal-shaped robot with the major senses such as AI and NUL based vision, hearing and balance. Paro interacts with users through speech and behavioral pattern recognition, and can express emotions according to the situation	Participants communicated freely with Paro at the desired time from morning to afternoon for about 2 months. POMS indicators and urine tests were conducted to measure participants’ emotional and physiological responses	The results showed that Paro encouraged communication, strengthened social bonds, and had a significant effect on emotional stability. In particular, the urine tests showed positive changes in hormone levels associated with physiological responses	•	•	•
Libin and Cohen-Mansfield, 2004	Nine elderly people aged 83–98 at the dementia center	NeCoRo, a cat with synthetic fur, can recognize human behavior and the environment through internal sensors of tactile, sound, visual and direction. Using 15 actuators inside the body, actions are created according to internal emotions	Participants communicated freely with NeCoRo for about 30 min, and indicators such as ABMI, LMBS, and AAID were utilized to measure cognitive function and behavioral disorder levels.	The results showed that the more severe the symptoms of dementia, the lower the level of interaction. However, the participants’ physically disruptive behavior and overall agitation decreased significantly, and they felt happier following the interaction with the cat	•	•	
Cornejo et al., 2013	Two extended families, including an elderly person who has never used a computer or a smartphone	Tlatoque is a digital frame that provides a means to communicate with Facebook to exchange images and other information collected by users in their homes. That is, it is a ubiquitous system that moves general SNS functions out of the desktop.	Most family members lived away from the elderly at the time of the experiment. Additional interviews with senior citizens were conducted to investigate subjective responses, along with the information collected from Tlatoque, which was placed in each household for a total of 21 weeks.	Tlatoque was not only easy to use but also facilitated the integration of SNS and was convenient to use in the information exchange process. All participants were positive and enthusiastic about using Tlatoque leading to enhanced family interactions offline.		•	•
Angelini et al., 2015	Participants in the demonstration event for the prototype	The study developed a prototype of “Multisensory Interactive Window” for the elderly immersive experiences. It supports family connection and virtual tourism, and the intuitive interface allows users to interact with the target at anytime and anywhere	Through the opportunity to experience the prototype, the subjective opinion about the Multisensory Interactive Window, and the modification and supplementation on the way to output the 3D object, environment, voice, and fragrance were drawn	Empirical assessment validate the possibility of providing realistic and vivid interactions such as moving the camera to the center of the screen and creating a scent using an ultrasound humidifier through electrical plug control		•	•

*“∙” means there is a connection between rows and columns.*

However, the results of our analysis results indicate that research on immersion technology for the elderly is immature and is mostly based on experiments focused on the Internet and smartphone usage. It is important that immersion technology induces interaction with virtual reality, virtual information, and virtual objects and that it functions to immerse the user in the information or content provided.

Hence, we can say that there is a need for the adoption of major new technologies such as visual reality (VR), augmented reality (AR), AI, NLU, and holographic reality (HR), in order to enhance the immersive experience for the elderly.

Recently, the immersive experience services have been successful at providing products and services in various fields, such as entertainment, education, and medical care. Moreover, with the advancement of major technologies, these are also being used in a more complex manner. This study investigated trends in immersive technology-based products and services that support successful aging. Priority was given to the immersive technology-based products where the customer base or target audience was elderly or where the product and service objectives could function for successful aging. The specific case analysis referred to the information provided by the official homepage of the product and service provider, based on the fact that the official homepage is the best medium to provide clear and detailed information on the relevant product. The products and services based on immersive experience technology for the elderly are presented in Table 5.

**TABLE 5:** Product and services used in immersive experience technology.

Technology	Service/product	Source	
VR	Step-by-step experience content for lifelong education and rehabilitation	Ericsson-LG	https://www.ericsson.com/en/future-technologies
	VR/AR content for the elderly memories and natural recreation experiences	Rendever	https://rendever.com/
VR/AR	Remotely controllable virtual olfactory device “VAQSO VR”	VAQSO	https://vaqso.com/
AR	AR projector lamp “Komorebi” to create virtual sunshine and shadows	LESLIE NOOTEBOOM	https://leibal.com/products/komorebi/
	Artificial light window that provides virtual sunlight and sky	CoeLux	https://www.coelux.com/en/home-page/index
	Skype lounge to interact with friends and family through immersive displays and audio	Maplewood Senior Living	https://www.maplewoodseniorliving.com/
	AR indoor navigation app “Gatwick Airport Advertisement” at London’s Gatwick Airport	Gatwick Airport	https://www.gatwickairport.com/
	Qualcomm immersive Audio (3D sound)	Qualcomm	https://www.qualcomm.com/invention
AI/NLU	KIA Motors’ Real-time Emotion Adaptive Driving (R.E.A.D. System)	Kia Media	https://www.kiamedia.com/
	Social robot PARO for communication and emotional care	AIST	http://www.parorobots.com/
	AI-based fall prediction and detection device	Kardian	http://kardian.com/
	Social speaker NUGU providing natural language (speech) recognizing daily life services	SKT	https://www.nugu.co.kr/
	AI button MAGO, which provides services such as daily activities services, dialogue and emergency treatment	MIKAWAYA21	http://pr.mago-btn.com/
HR	Hologram table (Holoamp)	Holoamp	http://hololamp.tech/
	Hologram projector device	Studio joanie lemercier	https://joanielemercier.com/
	“HealthVoyager” an immersive medical opinion system at Boston Children’s Hospital	HealthVoyager	https://www.healthvoyager.com
	HoloLens for telemedicine and real-time communication	Microsoft	https://www.microsoft.com/en-us/hololens

Virtual reality and AR technologies focus on health benefits that could be achieved through the provision of an immersive positive experience with nature. Examples include a lamp to project virtual sunshine designed by Leslie Nooteboom, a design engineer in England, and an artificial skylight to provide the experience of sunshine and sky proposed by Komorebi and CoeLux, a company for developing light systems. The startup company Rendever in the United States provides a step-by-step solution for education and recuperation of the elderly community, as well as a platform that allows users to experience a virtual natural environment. Maplewood Senior Living Center in the United States, which receives Rendever’s services, is actively adopting VR/AR technology, including a creation of an immersive “Skylounge.”

The AI/NLU technology can provides services to the elderly in everyday life and emergencies, based on natural language recognition for smooth communication and learning from the collected data. In a related case, Japan recently developed an animal-shaped robot as a solution to address the isolation and loneliness of the elderly; the robot provides user benefits such as communicating with animals and being comforted without having to look after a real pet (Banks et al., 2008). Paro, developed by the National Institute of Advanced Industrial Science and Technology (AIST) in Japan, is a seal-shaped robot that consists of four main senses: AI-based and NLU-based vision, hearing, balance, and touch. The assessment of the benefits of its use showed that the psychological status of the elderly was improved to a level similar to that of living with puppies (Wada and Shibata, 2007).

Other examples include Sony’s Aibo and Omeron’s NeCoRo, which have similar functions and purposes. In addition, KIA’s Real-time Emotion Adaptive Driving (READ System), presented at the Consumer Electronics Show (CES) in 2019, could recognize the driver’s vital signs and control the five senses in the vehicle to optimize the internal space of the vehicle considering a driver’s emotions and situation in real time. In other words, current AI technology can adjust cameras and various sensors controlling music, temperature, lighting, and aroma based on user emotions and surrounding conditions.

Finally, HR technology is a major technology that can maximize visual immersion and create virtual objects that can interact with the physical environment in 3D (Buckley, 2011). Advances in screenless display technologies have led to the development of projector-based hologram tables and hologram projectors. The “HealthVoyager,” an immersive medical observation system at Boston Children’s Hospital, can provide medical care in 3D for young patients and caregivers. In addition, the HoloLens, developed by Microsoft, also provides 3D virtual objects that can interact with users in real time. Medical science is exploring how to use HoloLens to improve our understanding of telemedicine and care (Sirilak and Muneesawang, 2018). As such, the development and use of immersive experience technology suggest that environmental elements such as color, texture, and temperature/humidity can function as immersive tools. This makes it essential to be used to support our aging society.

## Immersive Experience Service Model in the Elderly Welfare Center

### Framework of Immersive Experience Service

**FIGURE 2:**
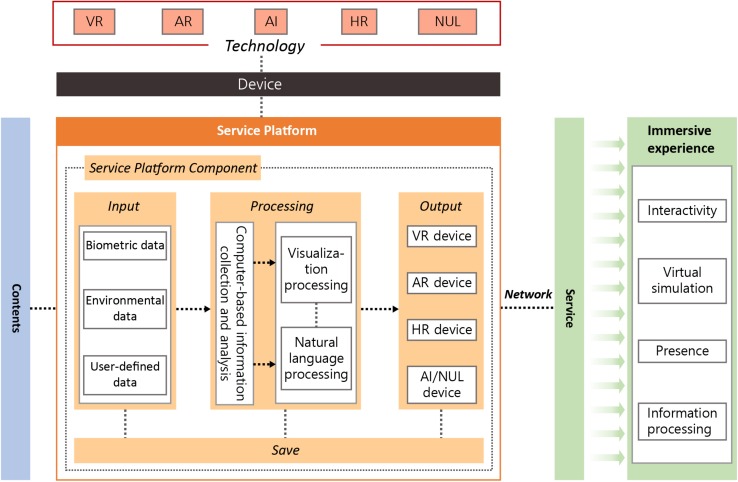
Framework of the immersive experience service.

The immersive experience service is an ecosystem-based industry where contents, a service platform, network, and a device are all combined. The service requires an open platform to implement the related technologies. The service platform consists of components with individual functionalities, such as “input” to collect data, “process” to informationize and process the data, “store” to store the collected data and processing results to reuse them, and “output” to understand and share the characteristics of the results data. Figure 2 presents the framework of the immersive experience service proposed in this study.

The framework of the immersive experience service for successful aging informationizes the input data, synthesizes the processed result, and sends it to the device that realizes the service according to the program content, in order to support successful aging. The user can experience immersion via the provided service. Components and content of the immersive experience service platform for successful aging are as follows. The input data include biometric information, environmental information, user-specified information, and so forth. The data input from a user or an administrator, such as program information provided by a welfare facility or user-specific matter, is user-specified information. The input data are collected for the analysis or analyzed according to predefined principles and then visualized or natural language processed to produce a result. Each collected datum and result are archived for reuse and serviced through a connected output device.

### Immersive Experience Service Platform for Successful Aging

Table 6 shows an immersive experience service platform for successful aging.

**TABLE 6:** Immersive experience service platform for successful aging.

Successful aging	Immersive experience service		Input	Processing	Output
Disease prevention	S.1	Remote virtual medical service	User-definition, Environment	HR graphic	HR
	S.2	Immersive medical note service based on the health conditions	User-definition, Biometric	HR graphic	HR
	S.3	Diet proposal and food information provision based on the health condition	Biometric, User-definition	AR graphic	AR
	S.4	Identification of those at risk of falls and injuries, and undertaking preventive measures by learning various postures	Biometric, Environment, User-definition	AI program, NLU program	AI, NLU
Physical and recognitive functionality	S.5	Step-by-step AI-based virtual training contents	Biometric, User-definition	VR graphic	VR
	S.6	Step-by-step virtual training contents based on the physical capability	Biometric, User-definition	VR graphic	VR
	S.7	Real-time route detection and AR navigation service based on the program	User-definition, Environment	AR graphic	AR
Psychological characteristics	S.8	Providing scattering of light, sunshine, and virtual sky through illumination	User-definition	AR graphic	AR
	S.9	Virtual travel contents for natural resorts or tourist attractions	User-definition	VR graphic	VR
	S.10	Virtual nature environment factor (indoor garden, aquarium, etc.) service in space	User-definition	AR graphic, HR graphic	AR, HR
	S.11	Customized indoor environment (lighting, sunlight, sound, etc.) service based on the physical/emotional status	Biometric, Environment, User-definition	AI program, NLU program	AI
	S.12	Virtual memory remembrance contents based on the experience	User-definition	VR graphic	VR
Productive activity	S.13	Customized job information service through analyzing data on training and learning	User-definition	AI program, NLU program	AI
	S.14	Virtual job experience and education contents	User-definition	VR graphic	VR
	S.15	Customized virtual environment contents associated with hobbies/education programs	User-definition	VR graphic, AR graphic	VR, AR
	S.16	Sharing hobbies and education outcomes with the elderly from other regional communities	User-definition, Environment	AR graphic, HR graphic	AR, HR
Social interaction	S.17	Realistic 3D virtual object (bird, dolphin, etc.) service	User-definition, Environment	HR graphic	HR
	S.18	Social robot service that recognizes and expresses natural language	Biometric, User-definition	AI program, NLU program	AI, NLU
	S.19	Immersive space service that allows to remotely communicate with friends and family	Environment, User-definition	AR graphic, HR graphic	AR, HR
	S.20	Multimodal participation virtual environment and game contents based on social VR	User-definition	VR graphic	VR

A total of 20 services were identified in this study. The input component data, according to each content item, are biometric data, such as user health information and emotional state; environmental data, such as temperature/humidity, direction, light, and pressure information; and user-specified information, such as the support program and structure, elderly learning level, and parental contact information. In particular, as successful aging is a multidimensional and complex concept, it is important to continuously manage the knowledge of experts and facility personnel in each field such as individual counseling contents, physical and cognitive ability levels, and family relationships, in order to support various characteristics of elderly welfare center users. That is, it is important to provide a customized immersive service through real-time update and input of user-specified information. Information processing can be divided into two groups: VR/AR/HR graphic processing for visualizing analysis based on each data and AI/NLU program for recognizing and expressing natural language with a predetermined learning pattern. When the service is ready to deliver via each processing method, it can be outputted to the connected VR/AR/HR/AI/NUL device.

We presented psychologically immersive experience services as a series of examples. From the perspective of successful aging, it is important to support psychological traits to provide a controllable environment and qualities that remind us of good experiences and memories (Baltes and Baltes, 1990; Bhatt, 2015). To this end, the immersive experience service implements a VR graphic based on personal experiences or memories and can provide access to a virtual environment (e.g., a childhood home) through a VR device (S.10 and S.12). Furthermore, it can provide an immersive environment that allows interaction with virtual nature elements (sunlight, sky, animals, plants, etc.) and can be adjusted through AR lighting and HR projector through an intuitive AR/HR graphic process (S.8 and S.10). Finally, vocal and facial expression pattern information is analyzed by the AI and NLU programs to provide a customized indoor environment through the autonomous control device (S.11).

### Immersive Experience Service Model by Space of an Elderly Welfare Center

Based on the derived immersive experience service, this study proposes an immersive experience service model by space of an elderly welfare center for successful aging, taking into account the functionality of an elderly welfare center. Table 7 shows an immersive experience service model based on an elderly welfare center.

**TABLE 7:** Immersive experience service model according to the elderly welfare center.

													Characteristic Immersive
Contents	Technology	Service	Function and spaces										experience
			Leisure and education			Function recovery		Counseling and employment		Promoting exchange			
						
			Hobbies		Physical	Medical						Communal	
			activity	Education	activity	care	Rehabilitation	Counseling	Employment	Refection	Rest	space	
Medical treatment	HR	S.1				O							Presence
	HR	S.2				O							Presence
Health information	AI/AR	S.3								O			Information processing, Interactivity
Prevention of falling	AI/NLU	S.4			O		O						Information processing
Cognitive training	VR	S.5					O						Interactivity, Virtual simulation
Physical training	VR	S.6			O								Interactivity, Virtual simulation
Navigation	AR	S.7	O	O	O	O	O	O	O	O	O	O	Interactivity
Natural healing	AR	S.8	O	O	O	O	O	O	O	O	O	O	Virtual simulation
	VR	S.9	O								O		Virtual simulation
	AR/HR	S.10									O	O	Presence
Mental stability	AI/NLU	S.11				O	O	O			O		Information processing
Recollection of memories	VR	S.12									O		Virtual simulation
Employment information	AI	S.13							O				Information processing
Hobbies and education	VR	S.14							O				Virtual simulation
	VR/AR	S.15	O	O									Virtual simulation
	AR/HR	S.16	O	O									Interactivity, Presence
Communion with nature	HR	S.17								O	O	O	Presence
Communication	AI/NLU	S.18								O	O	O	Information processing, Interactivity
	AR/HR	S.19									O		Presence
	VR	S.20			O								Virtual simulation, interactivity

*“O” means there is a connection between rows and columns.*

Immersive experience content and services are provided through successful aging and functionality of the center. As mentioned above, these can be applied to up to 10 spaces in these centers. Additionally, a user can experience immersion such as presence, interactivity, virtual simulation, and information processing depending on a service provided. To apply this service in each space, the lobby and educational spaces need to support customized virtual experience activity for each program based on VR/AR technology. It is also important to create an immersive multimodal participation-based learning environment by sharing the results with the elderly in other communities. The physical activity space should support enjoying the virtual activities together with the other elderly by using social VR. In an autonomous space, such as a fitness room, it is necessary to learn the behavioral patterns for fall and have virtual coaches for exercise equipment. In a medical space, remote treatment through a hologram is needed due to the absence of a specialist, and a medical note service is provided using a virtual object to help understand more about the health condition. The rehabilitation space needs to provide an autonomous environment adjustment, such as providing and recovering VR content step by step, in order to improve cognition and physical function, and light and temperature/humidity according to the emotional state. In the counseling and employment space, individual counseling and employment support should be provided based on the learning ability and aptitude of the elderly, and it will be important to build confidence in job activities following retirement, through virtual job experience. The elderly welfare centers providing lunch should develop a method to provide information in an easy way using an AR application, such as analyzing the health conditions and diet-related information of an elderly person to provide customized meals and health-related information through food photos.

The resting area is a center of immersive experience that can provide up to nine services. It can provide a dynamic nature immersion environment, such as a virtual sky, sunlight, HR/AR, and natural creatures (plants, birds, dolphins, and so forth), for psychological well-being and relaxation. Lastly, the public space in these centers is a space where exchange and movement occur. As the elderly welfare center program is conducted in different places depending on the individual timetables, it will be important to support navigation by providing an AR navigation tool that can guide the route in real time. In addition, an AI social robot that recognizes and expresses natural language can be used as a primary means of communication and provision of information.

## Discussion

This study proposed the immersive experience service model through linkage with immersion technology, considering the function and space composition of the elderly welfare center for the successful aging of the elderly. The architectural field has emphasized the improvement of physical environment or physical support focused on an evidence-based design, a universal design, and a barrier-free design. The purpose of this study was to explore the elderly welfare center environment from a new perspective by linking immersive experience skills that improve life satisfaction and social interaction, as a way to support mental and social functions important in successful aging. In addition, the immersive experience service discussed in this study can alleviate the physical and spatial constraints of the elderly and can function positively in successful aging by providing immersion into the desired environment and situation. Thus, it has sufficient value to support its application in the elderly welfare centers. The results of this study are summarized as follows.

First, successful aging is a process of actively coping with deterioration and impairment of old age, and can be defined as disease prevention, high level of physical and cognitive function, psychological stability, productive activity, and social relationships. Therefore, it is necessary to prepare support measures for successful aging in a comprehensive manner through linkage with local communities and by applying technology to efficiently provide this. In particular, the elderly welfare centers, as facilities used for the leisure and education of the elderly, recovery of skills, counseling and employment, and social interaction, must compose programs and provide relevant services to support successful aging. In this study, we analyzed the spatial composition of the elderly welfare centers by function to support successful aging by classifying these into hobbies, physical activity, education, medical, rehabilitation, counseling, employment information, refection, rest, and common spaces.

Second, the analysis of the previous studies shows that the effects of the immersion experience skills of the elderly are improved through improved physical balance and fatigue levels (Lai et al., 2013; Wu et al., 2013; Calogiuri et al., 2018), attention and memory (Zelinski and Reyes, 2009), and reduced depression, improved social skills, and stress reduction (Wu et al., 2013). This study derived the main technologies supporting the immersive experience of the elderly. These included VR, AR, AI, NLU, and HR. The immersive experience should be provided with the contents according to the functions of the user and the space as the main technologies are combined. Based on the results of our review examining the product and service trends of immersive experience technology, VR and AR technology include natural immersion product development, virtual training and rehabilitation content, and a path-finding application that induces a positive experience with nature. In this case, there are AI social robots for reducing the depression of elderly people through emotion recognition of facial expression and biosignal analysis and an autonomous control system for natural language communication. HR technology is developing products such as projectors and wearable devices to implement holograms, and medical finding systems and telemedicine are being increasingly used.

Third, the immersive experience service consists of content, platform, network, and device, and the service platform includes components of input, processing, storage, and output functions. This study constructed an immersive experience service platform for successful aging, with the input variables including biological information, environmental information, and personal information of elderly people. Processing collects and analyzes information about the input data and then visualizes or uses natural language processing to produce the result. The storage maintains the generated results for reuse, with the output being a device that presents the generated results to the outside world. The elderly are provided with the immersive experiences of presence, interactivity, virtual simulation, and information processing characteristics depending on the elderly welfare center’s available space and programs.

Finally, we derived 20 service content items considering the goals of successful aging and functions of the elderly welfare centers. We also suggested an immersive experience service model based on the functionally classified space of the elderly welfare center. The service content for each space is as follows. The hobbies and educational spaces support virtual experience activities according to the program and provide a multiparticipatory learning environment that shares the results with the elderly of other communities. In the physical activity space, virtual activities for each group should be performed according to the level of physical function, and in the case of an autonomous activity space without a monitor, a fall prediction system and a hologram virtual coach will be required for the safety of the elderly. As medical spaces in the centers are not manned, it will be important to support a good flow of communication with the elderly, such as delivering medical results using virtual objects during remote medical care and providing medical treatment using holograms such as HoloLens. Rehabilitation spaces need to support step-by-step learning content for cognitive and physical function training and provide a customized environment based on the physical and emotional states of the elderly. The counseling and employment space will provide consultation and employment information by analyzing the elderly learning data and aptitudes, as well as providing virtual job experiences and virtual education. Food and beverage spaces will need to provide food based on individual diets and other food-related information using AR applications, and rest spaces could support psychological well-being through the interaction with animals and plants using a virtual natural environment. Finally, public spaces could provide a real-time route search and an AR navigation according to the individual program timetables and also provide information on facilities for the elderly welfare centers and support natural communication using social AI robots.

## Conclusion

This study sought to apply immersive experience technology to the successful aging of the elderly. The immersive experience service was derived by linking the survey contents with the recently released immersive technology. The service of the elderly welfare centers for successful aging was presented as 20 applications methods by space, according to the functional classification of the elderly welfare centers. The study proposed the use of an immersive experience service system based on the characteristics of the immersive experience, according to service content. This study is a novel Contribution To The Field of elderly friendly architecture and construction. By subdividing the immersive experience service system according to the services provided by the existing elderly welfare centers, this study contributes to the improvement of applicability of the immersive experience service system to the real-life elderly welfare centers. Finally, this study provides a direction in which to expand the application of immersive experience technology and services for the elderly, including the entertainment, education, and medical fields. However, it should be noted that the immersive experience service model proposed in this study deals with the current program content and is limited to the currently developed technology. A further study is required to analyze the existing quantitative data in a clear and rigorous manner (including conducting in-depth interviews with the users) and to broaden the scope of the search criteria to alleviate the limitations on the use of immersion technology for the elderly. The ultimate goal of this study is to apply immersion technology that can interact with programs for each space in the physical environment, such as at the elderly welfare centers, assuming that the experience of immersion and the characteristics of the technology can function positively toward successful aging. This can be of value for the development of the elderly welfare centers.

## Data Availability Statement

All datasets generated for this study are included in the article/supplementary material.

## Author Contributions

EL and SP conceived and designed the research. EL collected and analyzed the information by reviewing the literature and investigating the case, and providing a substantial contribution to writing of the manuscript. SP provided the insights about the outline in the process of research progress, and also consistently examined, modified, and supplement the manuscript. Both authors interpreted the results and the meaning of the research, and approved the final manuscript.

## Conflict of Interest

The authors declare that the research was conducted in the absence of any commercial or financial relationships that could be construed as a potential conflict of interest.
